# Exercise upregulates copeptin levels which is not regulated by interleukin-1

**DOI:** 10.1371/journal.pone.0217800

**Published:** 2019-05-31

**Authors:** Milica Popovic, Katharina Timper, Eleonora Seelig, Thierry Nordmann, Tobias E. Erlanger, Marc Y. Donath, Mirjam Christ-Crain

**Affiliations:** 1 Clinic of Endocrinology, Diabetology and Metabolism, University Hospital Basel, Basel, Switzerland; 2 Department of Clinical Research, Medical Faculty, University of Basel, Basel, Switzerland; 3 Department of Biomedicine, University of Basel, Basel, Switzerland; 4 Clinical Trial Unit, Department of Clinical Research, University Hospital Basel, Basel, Switzerland; University of Houston, UNITED STATES

## Abstract

**Objective:**

Studies have suggested that arginine vasopressin (AVP) and its surrogate marker copeptin increase during exercise, independently of serum sodium and/or osmolality. In extreme cases, this can lead to runners-induced hyponatremia. Interleukin-1 (IL-1) increases during exercise and induces AVP in animal models. We here therefore investigate whether copeptin (a surrogate marker for AVP) increases upon exercise in young and healthy males, and whether this increase is regulated by IL-1.

**Design:**

In a randomized, placebo-controlled, double-blind, crossover trial in 17 healthy male volunteers, the effect of the IL-1 receptor antagonist anakinra on exercise-induced copeptin was compared with placebo.

**Methods:**

Participants exercised for one hour at 75% of VO_2max_ and were not allowed to drink/eat 6 hours before and during the study. Participants received either 100 mg of anakinra or placebo 1h before exercise. Blood was drawn at certain time intervals.

**Results:**

In both groups, copeptin levels were induced by 2.5-fold upon exercise (p<0.001), from 4.5–10.6 pmol/l in the placebo, and 4.3–11.3 pmol/l in the anakinra group, (p = 0.38). One hour after exercise, copeptin levels dropped to 7.7 and 7.9 pmol/l in the placebo and anakinra group, respectively (p = 0.58). The increase of copeptin levels was not explained by sodium concentrations.

**Conclusions:**

Exercise induces a continuous rise of plasma copeptin levels in healthy male volunteers independently of sodium levels and fluid intake. This increase is not regulated by the IL-1 pathway.

## Introduction

Copeptin (the C-terminal portion of pre-pro-vasopressin) is secreted together with arginine vasopressin (AVP) in equimolar amounts and serves as a surrogate marker for AVP which is difficult to measure.[1] Elevation in serum osmolality and reduction in blood volume are the classic stimuli for AVP and copeptin production.[2] Another important stimulus is stress and thus copeptin is increased in several acute diseases predicting outcome.[3–11] Additionally, several studies have shown an enhanced AVP production during exercise in different settings, whereby the elevation in AVP levels correlated with the intensity of exercise.[12–18] Few studies have investigated exercise-induced copeptin dynamics. These studies have been performed either in an elderly population with comorbidities, in young volunteers in a hot environment, or under extreme conditions like an ultramarathon.[19–25] Moreover, all of these studies have measured copeptin levels only twice, before and after exercise. Although increasing osmolality and plasma volume loss seem to be potent drivers of AVP/copeptin production in exercising participants, this does not explain the whole effect: Three studies reported increased AVP and copeptin levels after an ultramarathon in spite of decreased sodium values and osmolality.[12,22,26] Furthermore, a recent study showed that at a sodium level of 143 mmol/L or a serum osmolality of 295 mOsm/kg, respectively, led to an absolute increase in copeptin of only 3 pmol/L, i.e. lower levels than achieved during exercise.[27] AVP levels also normalized after exercise without fluid replacement.[13,15] Therefore, other factors seem to be involved in the regulation of AVP/copeptin dynamics upon exercise. The pro-inflammatory cytokine interleukin-1 (IL-1) might play a role in AVP/copeptin production. Specifically, serum IL-1β levels and IL-1β activity were shown to increase upon exercise.[28–30] Furthermore, animal studies reported increased AVP production after administration of IL-1β in freely moving rats *in vivo* or in the dissected hypothalamus *in vitro*.[31–33] The fact that administration of dexamethasone abolished the increase in AVP levels in exercising healthy men further supports the inflammatory hypothesis in exercise-stimulated AVP production.[34]

The aim of this study was therefore first to investigate the course of copeptin before, during, and after moderate exercise in a young and healthy male population. Second, we aimed to test whether IL-1 regulates exercise-induced copeptin.

To address these questions, we evaluated copeptin levels before, during and after aerobic exercise in 17 healthy male participants and the response of copeptin to either the IL-1 receptor antagonist anakinra or placebo. Furthermore, we measured sodium levels at the beginning, the end, and 1 hour after the end of an exercise bout.

## Participants and methods

### Study design

This is a post-hoc analysis of a randomized, placebo-controlled, double-blind, crossover, and single-center clinical study.[35] Patients were recruited and followed-up from November 2011 to May 2013 at the University Hospital Basel, Switzerland. Informed consent was obtained from all participants prior to study inclusion. Participants were treated with either 100 mg of the IL-1 receptor antagonist anakinra or placebo. The study was performed in accordance with the ICH-GCP guidelines and the Declaration of Helsinki, was approved by the Ethics Committee of Basel on March 21^st^, 2011 (Ref. 294/10) and Swissmedic (Ref. No. 2011DR1084), and was registered on clinicaltrials.gov (NCT01771445). As the original study was a mechanistic and not a treatment study, it was realized with a delay of 3 months that the study had to be registered. The authors confirm that all ongoing and related trials for this drug/intervention are registered.

### Eligibility criteria

Eligible participants were male, apparently healthy, non-smoking, aged between 20 and 50 years with a body mass index between 18 and 26 kg/m^2^. Participants had to exercise regularly prior to study inclusion. Exclusion criteria were any evidence of acute or chronic illness, history of carcinoma or tuberculosis, increased alcohol consumption, known allergy to anakinra, and current treatment with any drug. Furthermore, participants were excluded if they had used anakinra within 30 days prior to enrollment.

### Treatment assignment and blinding

After successful inclusion in the study, participants were assigned a random subject number to receive study medication. Treatment blinding and preparation of trial drugs were performed by the Clinical Trial Unit of the University Hospital Basel, Switzerland.

### Study procedures

The exact study procedures were previously described.[35] Shortly, the study consisted of three visits, one screening visit in which VO_2max_ was determined, followed by two study visits that were separated by seven days. The VO_2max_ determination took place at least 7 days before the main study visits. After inclusion, patients were allocated according to a randomization list created by a biostatistician unrelated to the study. Patients as well as study personnel were blinded to the medication allocation. Participants were not allowed to drink or eat 6 hours prior to and during the two study visits. Sixty minutes prior to exercise start, an intravenous catheter was placed in the forearm, and the first blood sample was drawn. Immediately after taking the first blood sample, participants received a single subcutaneous injection with 100 mg of anakinra or placebo in a double-blind, crossover manner. At the beginning (0 minutes), the subject started to run on a treadmill with a 5 minutes warm up period at 2 to 4 km/h at an incline of 0.5%. The treadmill speed was then increased to 75% of VO_2max_ (which was previously determined) based on heart rate measurements for 60 minutes followed by a “cool down” period at walking speed for 5 minutes. A 0.9% saline infusion was maintained at very low rates to ensure venous catheter flow. Sixty minutes after the end of the exercise bout, the intravenous catheter was removed. In total, blood was drawn at 12 time points: 60 minutes before exercising (-60 minutes), every ten minutes during exercise starting immediately prior to the exercise until immediately after (0, 10, 20, 30, 40, 50, 60 minutes) and four times within the hour following the exercise (70, 80, 90, 120 min). The same procedures were performed one week after the first visit followed by a safety visit after an additional week.

### Sample collection and analytic procedure

Blood was collected into serum tubes, centrifuged at 4°C and aliquoted. Samples were frozen and stored at -80°C until measurement. The aliquot tubes have not been subject to refreezing before copeptin / sodium measurements. Copeptin was measured by an immunofluorescent assay (BRAHMS CT-proAVP KRYPTOR, Thermo Scientific Biomarkers, Hennigsdorf, Germany) in serum. Sodium values were measured by an ion-selective electrode system (cobas 8000, Roche Diagnostics GmbH) in serum.

### Statistical analysis

Summary statistics were performed and the course of mean copeptin values from 0 to 120 minutes plotted including 95%-confidence intervals for each measurement. It was assumed that between measurements concentration increases or decreases linearly. Hence, values of consecutive measurements were connected with a straight line.

To test 1) whether copeptin values change during time, 2) whether there is a difference between the placebo and the anakinra group at 60 and 120 minutes, and 3) whether the copeptin levels are explained by sodium concentrations, linear mixed-effects models were fit. Outcome variables were log-transformed copeptin levels (in the results section, original copeptin values are shown). Fixed-effect explanatory variables were time of measurement, treatment allocation, and sodium levels. Random-effect variable was the unique participant identifier. The three missing copeptin values of two participants and six sodium values of three participants were imputed with the median of the respective time of measurement and treatment. All calculations were performed using the imputed data set. Sensitivity analyses are done with the original data base containing missing values.

The area under the curve (AUC) for every exercise bout from 0 to 120 minutes was calculated using original values. Whether there was a significant difference of AUCs between copeptin values of anakinra and placebo treatment was tested with a Wilcoxon signed rank test.

## Results

### Baseline characteristics

The baseline characteristics of all participants have been previously published.[35] Briefly, all participants were male, mean age was 29 years, and all had a normal BMI. Blood pressure and heart rate were within the normal range in all participants. No subject showed evidence of any medical condition or signs of acute or chronic low-grade inflammation neither in clinical examination nor by laboratory assessment (e.g. measurement of C-reactive protein levels and leukocyte count).

### Copeptin levels upon exercise with and without IL-1 receptor antagonism

**Fig 1:**
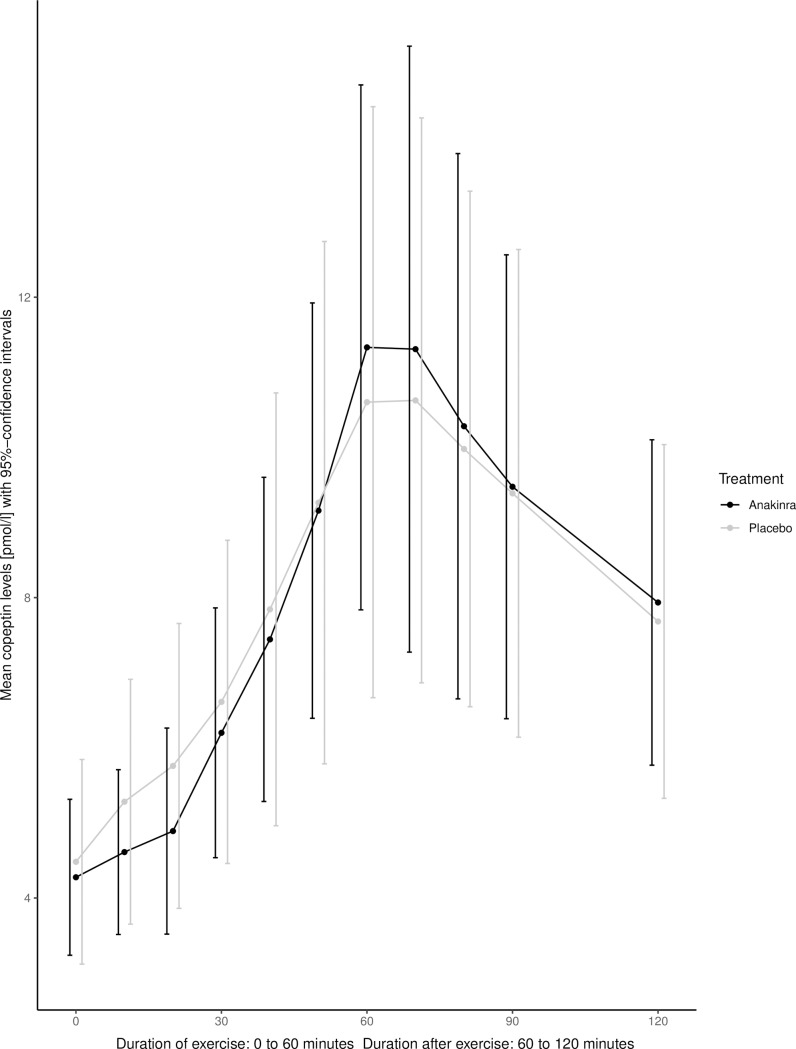
Dynamics of exercise-induced copeptin with and without IL-1 receptor antagonism.

Fig 1 shows the dynamics of copeptin levels during (0 to 60 minutes) and after (60 to 120 minutes) exercise. Before the start of the exercise bout, copeptin levels were 4.5 (SD: 2.7) pmol/l and 4.3 (SD: 2.0) pmol/l in the placebo and anakinra group, respectively. In both groups, copeptin levels rose significantly by 2.5-fold upon exercise and peaked at the end of the exercise period (60 minutes, p<0.001). At that time, copeptin levels were 10.6±7.7 pmol/l in the placebo group and 11.3±6.8 pmol/l in the anakinra group. No difference was observed between the two treatment groups at 60 minutes (p = 0.38). One hour after exercise, copeptin levels dropped to 7.7±4.6 pmol/l in the placebo group (p = 0.015) and 7.9±4.3 pmol/l in the anakinra group (p<0.1), showing no difference between the two treatments (p = 0.58, Table 1). In both groups, sodium levels rose from 141 (SD: 1.8) mmol/l at baseline to 143 (SD: 1.5) mmol/l at end of the exercise bout, while one hour after the end of the exercise period sodium levels were 141 (SD: 1.3) mmol/l (Table 1). Sodium levels did not modulate copeptin levels (p = 0.10). The AUCs of copeptin values for the time during and after exercise were not significantly different when participants were treated either with placebo or anakinra (p = 0.68). All the results mentioned were confirmed in the sensitivity analysis when using the original dataset without imputed values.

**Table 1:** Copeptin and sodium levels at different times.

Time of measurement (minutes)		0	60	120
Copeptin levels (pmol/l)	Anakinra group	4.3±2.0	11.3±6.8	7.9±4.2
	Placebo group	4.5±2.7	10.6±7.7	7.7±4.6
Sodium levels (mmol/l)	Both groups	141±1.8	143±1.5	141±1.3

Data represent the mean and standard deviations.

## Discussion

To our knowledge, this is the first study reporting the course of copeptin levels before, during, and after steady-state exercise and investigating the potential role of IL-1 in copeptin regulation. We report two main findings. First, exercise induced a continuous rise of copeptin levels by about 2.5 fold in healthy male volunteers reaching a maximum level of ~11.0 pmol/l at the end of the exercise period. Copeptin levels remained elevated in the first minutes after discontinuing exercise and then dropped gradually within the following hour to a level of ~7.8 pmol/l which was 1.7-fold higher than baseline. Second, our data suggest that exercise-induced copeptin is not regulated via the IL-1 pathway.

Our results are in line with previous studies that have shown an elevation of copeptin levels upon exercise. Two studies have shown an increase of copeptin levels by 2–3 fold (peak copeptin level: 10.1 pmol/l) in elderly patients with comorbidities after short-duration high-intensity exercise.[20,24] One study has performed copeptin measurements in the context of high-intensity exercise in healthy volunteers and patients with major depression, showing a 2.8-fold (peak 13.4 pmol/l) and a 1.9-fold (peak 9.7 pmol/l) increase in copeptin, respectively.[23] Another study investigating medium intensity exercise in a warm environment showed 2.6-fold copeptin increases (peak 14.3 pmol/l).[25] Lippi et al. reported a 6.4-fold increase in copeptin in healthy males participating in an ultramarathon (peak levels were 40 pmol/l).[21] Consistent with these findings, Aakre et al. reported a 3.5-fold elevation of copeptin in middle aged males after a bike endurance race (peak 12.8 pmol/l).[22] Similarly, several studies have investigated AVP levels in healthy exercising participants. All found an elevation of AVP levels depending on exercise intensity. Although in some studies increasing plasma osmolality, plasma volume loss and sodium levels correlated with AVP levels after exercise, other studies found an elevation of AVP levels independent of plasma sodium, osmolality, and fluid intake.[12–16,18] Consistent with these previous reports, in our study copeptin levels continuously increased during exercise and then dropped after the end of the exercise period, although participants were not allowed to replace the amount of fluid loss, and although serum sodium levels remained in the normal range at the end of the exercise bout. The drop in copeptin levels was consistent with findings on the course of AVP levels from the study by Wade and Claybaugh.[15] They investigated AVP levels in a similar setting of exercise intensity and duration. In that study AVP levels increased 2.2-fold at the peak of exercise. 60 minutes after end of exercise AVP levels dropped but remained 1.6-fold elevated compared with baseline. This is in concordance with our findings of a 1.7-fold elevation of copeptin levels 60 minutes after end of exercise.

Our data therefore suggest that the rise in copeptin levels upon exercise is not driven by increasing sodium levels and that other factors, such as exercise-induced inflammation, may play a role in AVP/copeptin production. Injection of the potent inflammatory cytokine IL-1β in the hypothalamus induced secretion of AVP in rodent models.[31–33] Interestingly, IL-1β is not only secreted during infection, but also during exercise.[28–30] Although the data on IL-1β elevation are controversial, it is important to note that measurement of IL-1β is not reliable due to minimal concentrations in the blood and only antagonization of the IL-1β can provide confirmatory answers on IL-1β effects.[36] Therefore, IL-1β might represent a valuable candidate driving exercise-induced AVP and copeptin levels. If so, then IL-1 receptor blockade would inhibit the exercise-induced increase in copeptin levels. However, copeptin levels in our study increased to the same extent in volunteers treated with an IL-1 receptor antagonist or with placebo. This suggests that exercise-induced copeptin is not regulated by IL-1 and that other factors that are activated during exercise (e.g. activation of the sympathetic nervous system, increase in body temperature, pain, nausea, IL-6) are responsible for driving the increase in AVP and copeptin upon exercise.[26,37–39] Nevertheless, the participants in this study did not report pain or nausea.

This study has several limitations. First, this was a post-hoc analysis which bears the risk of a type 2 statistical error. Nevertheless, a study by Coiro et al. using a similar study design showed significant reductions in peak AVP levels after anti-inflammatory treatment with dexamethasone in only 10 participants compared with n = 17 in this study.[34] Furthermore, the imputation of missing data points and comparisons of individual AUCs render a type 2 statistical error unlikely. Another limitation is that we did not measure levels of AVP. AVP has different decay kinetics than copeptin, i.e. a 2x shorter half-life, and our results can therefore not be directly extrapolated to AVP. Measurement of AVP could have revealed further insights into possible dissociations between copeptin and AVP. Lastly, we did not measure serum osmolality, but only serum sodium levels.[18,40]

Taken together, we here confirm an effect of exercise on copeptin levels that is independent of sodium levels or fluid loss. This finding suggests that it may be important to consider exercise as confounding factor when interpreting copeptin levels as a diagnostic and prognostic marker.[41] For example, patients should be asked not to do exercise before routine evaluation of polyuria polydipsia syndrome.

In conclusion, exercise induces plasma copeptin levels independent of sodium levels or fluid loss and not regulated by the IL-1 pathway.

## Supporting information

S1 File Individual copeptin values of the study participants. Copeptin values are given in pmol/l. The second page of this file contains a codebook explaining abbreviations.
(XLSX)

S2 File Individual sodium values of the study participants. Sodium values are given in mmol/l and were measured at the following times: Start and end of exercise, and one hour after exercise.
(CSV)

S3 File R-Code for statistical analysis. (R)
